# Discovery of Posture Secret manual therapy for post-stroke shoulder pain: study protocol for a randomized controlled trial

**DOI:** 10.3389/fneur.2025.1594560

**Published:** 2025-09-12

**Authors:** Chengning Song, Bo Lei, Zhixian Li, Haiyan Li, Songting Tian, Nana Feng

**Affiliations:** Department of Rehabilitation Medicine, Fuyong People's Hospital of Bao'an District, Shenzhen, China

**Keywords:** Discovery of Posture Secret, Standardized Chinese Tuina, post-stroke shoulder pain, musculoskeletal ultrasound, randomized controlled trial

## Abstract

**Background:**

Post-stroke shoulder pain (PSSP) is a frequent complication that significantly impedes upper limb rehabilitation in stroke patients and contributes to global healthcare costs amounting to billions of dollars each year. Beyond the financial impact, PSSP severely compromises patients’ quality of life. Developing effective treatments to reduce pain and improve shoulder joint function in individuals with PSSP remains a pressing clinical priority. We are currently conducting a rigorously structured randomized controlled trial to evaluate and compare the effectiveness of Discovery of Posture Secret (DPS) therapy and Standardized Chinese Tuina Therapy (SCTT) in stroke patients diagnosed with shoulder pain through musculoskeletal ultrasound. This paper presents the trial’s design and implementation.

**Methods/design:**

This is a single-center, 4-week randomized controlled trial carried out at the Department of Rehabilitation, Fuyong People’s Hospital, Bao’an District, Shenzhen, Guangdong Province. The objective is to assess the comparative effectiveness of DPS and SCTT in patients with PSSP. Eligible participants are stroke survivors aged between 40 and 60 years who meet the diagnostic criteria for PSSP established in China. Participants will be randomly assigned to one of two treatment groups, receiving one 40 min session each weekday (Monday through Friday) for four consecutive weeks, with rest on weekends. The primary outcome is the change in pain intensity measured by the Visual Analog Scale (VAS) at 2 weeks, 4 weeks, and at a 1-month follow-up. Secondary outcomes include shoulder joint function and general functional recovery at 4 weeks, evaluated using shoulder range of motion (ROM), the absolute difference in vertical distance between the medial superior angle of the scapula and the second thoracic spinous process, musculoskeletal ultrasound findings, and the Modified Barthel Index (MBI).

**Discussion:**

This is the first randomized trial to compare the effectiveness of DPS and SCTT in PSSP patients, with a one-month follow-up. We propose a carefully constructed randomized controlled trial that, for the first time, employs musculoskeletal ultrasound as an objective tool for assessing PSSP. This methodological advance aims to increase the objectivity and reliability of the outcome data. The results of this trial are expected to carry substantial public health relevance for the growing population affected by PSSP and may inform better treatment approaches and functional recovery strategies.

**Clinical trial registration:**

ClinicalTrials.gov, identifier NCT06763796.

## Introduction

PSSP is a common complication after stroke, characterized by pain in the shoulder region, typically on the hemiplegic side. It stems from both musculoskeletal and neurological dysfunctions and significantly hampers functional recovery (1). Clinically, PSSP presents with localized or referred pain, limited range of motion, muscle spasticity, and, in some cases, shoulder subluxation (2). Prevalence estimates vary widely, from 16 to 84% among stroke survivors, depending on diagnostic criteria and study populations (3). The condition often leads to prolonged hospitalization and imposes a heavy financial toll, contributing to billions in global healthcare expenditures annually, driven by direct medical costs and lost productivity (4). Clinical reports further suggest that PSSP affects not only postural stability and mobility but also delays recovery of hand function and complicates basic self-care tasks, increasing the caregiving and financial burden on families (5, 6). These factors point to an urgent need for effective treatments that can reduce pain and support rehabilitation progress.

Currently available therapies for PSSP face serious limitations in terms of efficacy, accessibility, and safety. Nonsteroidal anti-inflammatory drugs (NSAIDs), such as celecoxib, are frequently unsuitable for patients with cardiogenic stroke, particularly those who have undergone cardiac surgery, due to the elevated risk of serious cardiovascular thrombotic events, including myocardial infarction and stroke, especially in those with existing cardiovascular disease (7). Neural blockade, though potentially effective, requires ultrasound guidance and skilled practitioners, restricting its use to advanced healthcare settings (8). Physical therapy options like transcutaneous electrical nerve stimulation (TENS) and electroacupuncture contribute to pain control, but they carry risks for patients with implanted cardiac devices, such as pacemakers or defibrillators, owing to the danger of electromagnetic interference and device malfunction (9, 10). These challenges highlight the urgent demand for treatment strategies that are both low-risk and cost-effective, making this area a key focus in ongoing clinical research.

The DPS technique, developed by Professor Sang-Hee Won, a senior physical therapist from Korea with over two decades of clinical experience, and Professor Qiang Wang, Director of the Rehabilitation Medicine Department at Qingdao University Affiliated Hospital, introduces a novel joint movement method known as “shear motion.” This approach has demonstrated promising therapeutic effects in pain relief and neurorehabilitation (11–13). DPS begins with a structured postural assessment performed in the patient’s neutral stance to identify primary contributors to joint misalignment. Its foundation lies in the 4R technique: Resetting Joint Malalignment (RJM) corrects abnormal joint alignment; Resetting Abnormal Muscle Function (RAM) restores muscle performance; Resetting Joint Stabilization (RJS) re-establishes joint stability; and Resetting Sensory-Motor Control (RSMC) targets improvements in sensory and motor regulation. These combined interventions offer a multi-faceted strategy for correcting joint dysfunction and reestablishing functional movement.

In our clinical experience, supported by patient feedback, the DPS method has shown a clear capacity to reduce post-stroke shoulder pain, improve shoulder range of motion, and promote functional recovery of the hemiplegic limb. Encouraged by these observations, we have initiated a large-scale trial to assess the comparative effectiveness of DPS in patients experiencing symptomatic post-stroke shoulder pain. We hypothesize that DPS will demonstrate greater efficacy than SCTT. The goal of this study is to evaluate DPS as a potentially more effective treatment that not only reduces pain but also improves shoulder mobility and minimizes functional impairments that affect the quality of life in millions of stroke survivors with hemiplegia.

This paper outlines the design and protocol of the first randomized controlled trial directly comparing DPS with SCTT for managing post-stroke shoulder pain. The study aims to fill an important evidence gap and offer practical guidance for clinical treatment of this complex condition. Results will be published in compliance with the Consolidated Standards of Reporting Trials (CONSORT) guidelines (14), ensuring transparency and methodological soundness.

## Methods/design

### Study design overview

**Figure 1:**
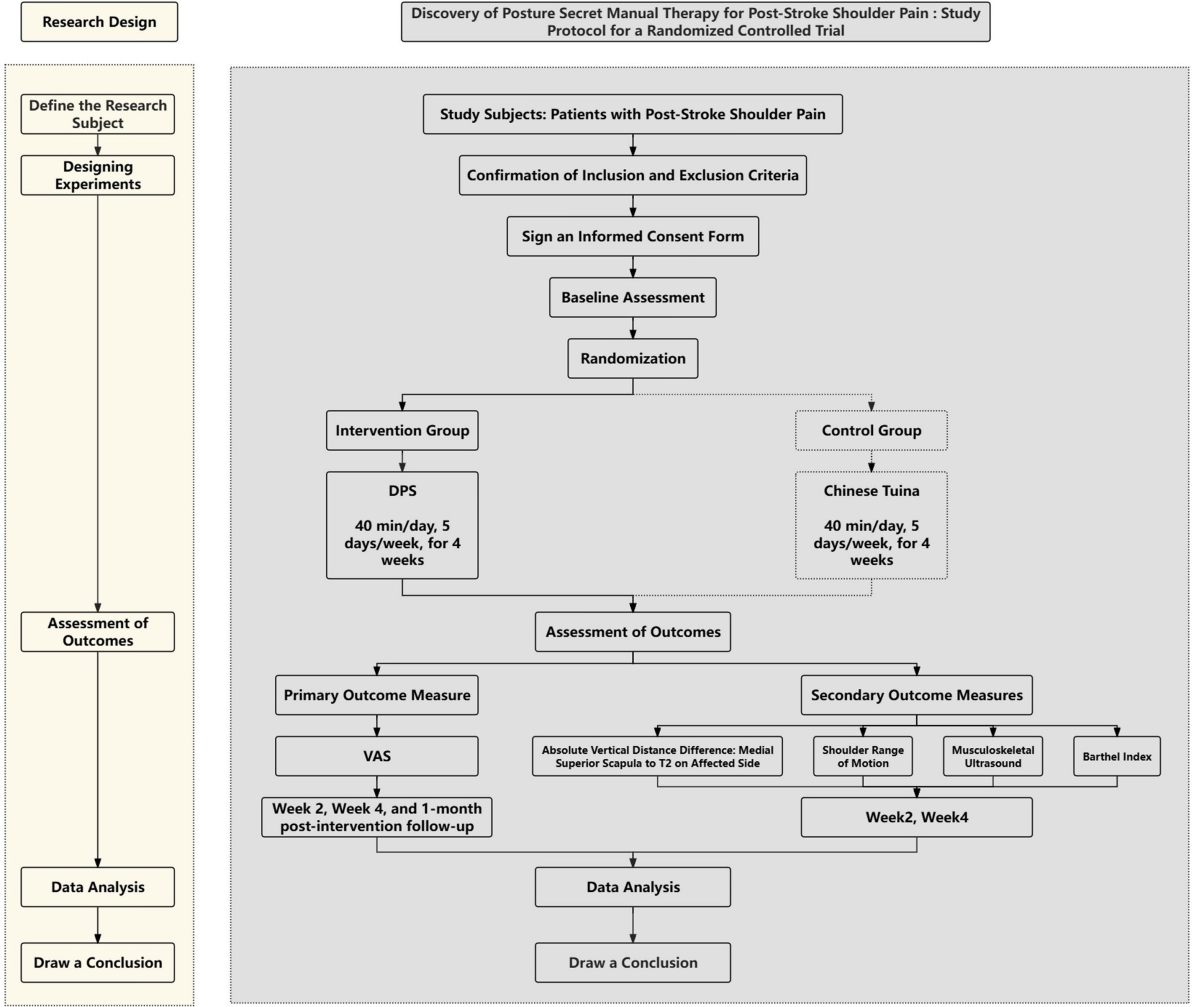
Study design flowchart: a detailed depiction of participant progression through each stage of the study.

This study is a 4-week, single-center, single-blind randomized controlled trial. Over the next 3 years, 48 patients diagnosed with post-stroke shoulder pain will be randomly assigned to either the DPS group or the SCTT group. Both treatments will be delivered once daily from Monday to Friday (five sessions per week), with each session lasting 40 min, over a four-week period. Weekends will be reserved for rest. Figure 1 presents the study timeline and intervention phases.

The primary outcome is pain intensity, assessed using the VAS, a widely recognized and validated tool. VAS scores will be recorded at baseline, at 2 and 4 weeks during treatment, and again at a 1-month follow-up after completion of the intervention. Secondary outcomes assess shoulder joint function across four domains. First, ROM will be measured to evaluate mobility of the affected shoulder. Second, the absolute vertical distance difference between the medial superior angle of the scapula on the affected side and the second thoracic spinous process will be recorded. Third, musculoskeletal ultrasound will be used to assess five key indicators: presence of effusion around the long head of the biceps tendon sheath, calcification in the supraspinatus muscle, calcification at the insertion of the subscapularis tendon, subdeltoid bursa thickness (in millimeters), and the distance from the acromion to the greater tuberosity of the humerus. Lastly, the MBI will be used to evaluate overall independence in daily living activities. Covariates include age, sex, body mass index (BMI), stroke type (hemorrhagic or ischemic), and time since stroke onset. All outcome measures will be collected at baseline, 2 weeks, and 4 weeks after treatment begins. A follow-up VAS will be conducted 1 month post-intervention to assess the sustained effects of DPS in relieving post-stroke shoulder pain. The physical function assessments will be performed by trained evaluators blinded to treatment allocation, and data analysis will be conducted by statisticians also blinded to group assignments.

Detailed outcome variables and their classification as endpoints or intermediate measures are summarized in Table 1.

**Table 1:** Baseline assessment and sequence of trial measurements for primary and secondary outcomes.

	Before-intervention	Mid-intervention	Post-intervention	1-month follow-up
Time (weeks)	**0**	**2**	**4**	**8**
Baseline				
Sex (male/female)	**√**			
Type of stroke (ischemia/hemorrhage)	**√**			
Age, y	**√**			
BMI	**√**			
Time since stroke, y	**√**			
Primary outcome variable				
VAS	**√**	**√**	**√**	**√**
Secondary outcome variables				
Shoulder ROM				
Flexion (0–180°)	**√**	**√**	**√**	
Extension (0–50°)	**√**	**√**	**√**	
Abduction (0–180°)	**√**	**√**	**√**	
Adduction (0–40°)	**√**	**√**	**√**	
Internal rotation (0–70°)	**√**	**√**	**√**	
External rotation (0–90°)	**√**	**√**	**√**	
Horizontal abduction (0–45°)	**√**	**√**	**√**	
Horizontal adduction (0–135°)	**√**	**√**	**√**	
Absolute vertical distance difference: medial	**√**	**√**	**√**	
Superior scapula to T2 on affected side				
Musculoskeletal ultrasound				
Distance from the acromion to the greater tuberosity of the humerus	**√**	**√**	**√**	
Effusion of the long head of the biceps tendon	**√**	**√**	**√**	
Calcification of the supraspinatus muscle	**√**	**√**	**√**	
Calcification of the subscapularis muscle	**√**	**√**	**√**	
Thickness of the subdeltoid bursa	**√**	**√**	**√**	
Modified Barthel Index	**√**	**√**	**√**	

“√” Indicates that the corresponding outcome will be assessed at that time point.

The study will take place in the Rehabilitation Department of Fuyong People’s Hospital, Bao’an District, Shenzhen, Guangdong Province, China. Ethical approval was granted by the Institutional Review Board of Fuyong People’s Hospital, Bao’an District, Shenzhen.

### Sample size calculation

This study draws on findings from a previously published clinical observational trial on the application of DPS for post-stroke shoulder pain (15). Sample size estimation was performed using G*Power 3.1 software. The significance level (*α*) was set at 0.05, *β* at 0.20, and statistical power (1−β) at 0.80. Based on the method for comparing two groups of equal size and using an effect size (Cohen’s d = 0.91) as reported in the referenced study, the minimum required number of participants was calculated to be 19 per group, totaling 38. To account for an expected dropout rate of 20%, the sample size was increased to 48 participants. This adjustment ensures sufficient statistical reliability and helps maintain the study’s validity despite potential participant attrition.

### Study sample

Participants will be eligible for inclusion if they meet all of the following criteria: (1) aged between 40 and 60 years; (2) diagnosed with a first-ever stroke (ischemic or hemorrhagic), confirmed by cranial CT or MRI; (3) onset of hemiplegic shoulder pain after the stroke, with a Visual Analog Scale (VAS) pain score ≥4; (4) Brunnstrom stage II to V on the affected limb; (5) no significant pre-existing shoulder disorders (e.g., rotator cuff tear, severe osteoarthritis, adhesive capsulitis) confirmed by ultrasound and clinical exam; (6) no severe cognitive impairment (Mini-Mental State Examination score ≥24); and (7) able and willing to provide written informed consent.

Participants will be excluded if they have: (1) subarachnoid hemorrhage; (2) two or more prior strokes; (3) significant shoulder subluxation (≥1 finger-width gap between acromion and humeral head) or flaccid paralysis; (4) clinical deterioration due to recurrent infarct or hemorrhage; (5) severe cardiopulmonary conditions (e.g., acute myocardial infarction, unstable angina, severe arrhythmia, aortic stenosis, pericarditis, malignant hypertension); and (6) other causes of shoulder pain unrelated to stroke, such as cervical spondylosis or referred visceral pain.

### Recruitment strategies

The recruitment plan for this study will rely on the existing resources and clinical collaborations within our hospital. The Rehabilitation Department includes 54 inpatient beds, with approximately 60% occupied by stroke rehabilitation patients, of whom nearly 80% experience post-stroke shoulder pain. This patient base provides a strong foundation for participant recruitment. Physicians, nurses, and physical therapists will actively engage eligible patients and inform them about the study to encourage enrollment. In addition, we have close working relationships with the Departments of Neurosurgery and Neurology, where patients are typically transferred to the Rehabilitation Department for further treatment about 10 days after the acute phase of stroke. This collaboration ensures access to a steady and suitable flow of potential participants.

Recruitment will also involve physician-led patient education, printed posters and advertisements placed in high-traffic hospital areas such as elevators and outpatient departments, and outreach through community healthcare centers to broaden awareness. These strategies are designed to secure the required sample size for the trial.

### Enrollment and the informed consent process

Participants will be enrolled in groups of at least 10 per intervention cycle to support a structured group intervention setting and maintain an equal 1:1 allocation between the two treatment arms. Within 2 weeks prior to the intervention phase, baseline assessments will be conducted for 10 to 20 pre-screened individuals to establish an eligible pool for randomization.

Before any data are collected, the principal investigator (CW) or the research coordinator will initiate the informed consent process. Each participant will receive a written consent form and a detailed explanation of the study. Those who agree to participate will sign the form. Following consent, eligibility will be confirmed based on the predefined inclusion criteria. Once eligibility is verified, the study team will explain the trial procedures in detail to ensure that participants fully understand the nature of their involvement. Completion of these steps finalizes the enrollment process.

### Randomization

Once written informed consent was obtained and baseline assessments were completed, an off-site research assistant performed the randomization using SPSS version 27. Participants were randomly assigned to either the DPS therapy group or the SCTT group. To reduce potential allocation bias, stratified randomization was applied based on time since stroke (in years) and stroke type (ischemic or hemorrhagic).

To ensure allocation concealment, all information regarding group assignment, therapy schedules, and intervention locations was communicated solely through centralized telephone contact, managed by the off-site research assistant. This method kept other study personnel blinded to group assignments and helped minimize bias throughout the intervention phase.

### Study intervention

Both the DPS therapy and SCTT were implemented concurrently to reduce variability caused by seasonal changes in symptom severity. During the four-week intervention phase, participants in both groups received manual therapy according to their assigned treatment. Each group underwent five therapy sessions per week, each lasting 40 min, for a total of four weeks. Participants remained in inpatient rehabilitation during this period, and weekly consultations were held to monitor adherence. The four-week treatment window reflects standard practice in China, where medical insurance typically limits inpatient rehabilitation to this duration.

Participants were instructed not to take nonsteroidal anti-inflammatory drugs (NSAIDs), including celecoxib, or acetaminophen during the trial. Medications such as celecoxib may pose risks for post-stroke shoulder pain patients with a history of cardiac stent placement, as they can impair platelet function and increase cardiovascular risk (7, 16). In cases of severe pain, acupuncture was used as a temporary alternative, integrated into the basic rehabilitation regimen. Routine rehabilitation therapy continued throughout the study to ensure uniformity in care delivery.

### DPS intervention

DPS therapy is composed of two core elements: assessment and treatment. The process begins with a posture assessment, which forms the basis of the intervention. Visual observation is first used to evaluate whether the trunk and limbs are in a neutral alignment. This is followed by palpation and movement testing to identify any joints or muscle groups that deviate from their neutral positions. The assessment includes a full postural evaluation of the trunk and the shoulder complex in a neutral stance. A distal-to-proximal approach is applied, examining the lumbar spine, thoracic spine, and both cervical and shoulder regions across the coronal, sagittal, and transverse planes. The shoulder complex is then assessed on both sides, focusing on the sternoclavicular joint, acromioclavicular joint, scapulothoracic articulation, and glenohumeral joint to detect any asymmetry or structural abnormality.

**Figure 2:**
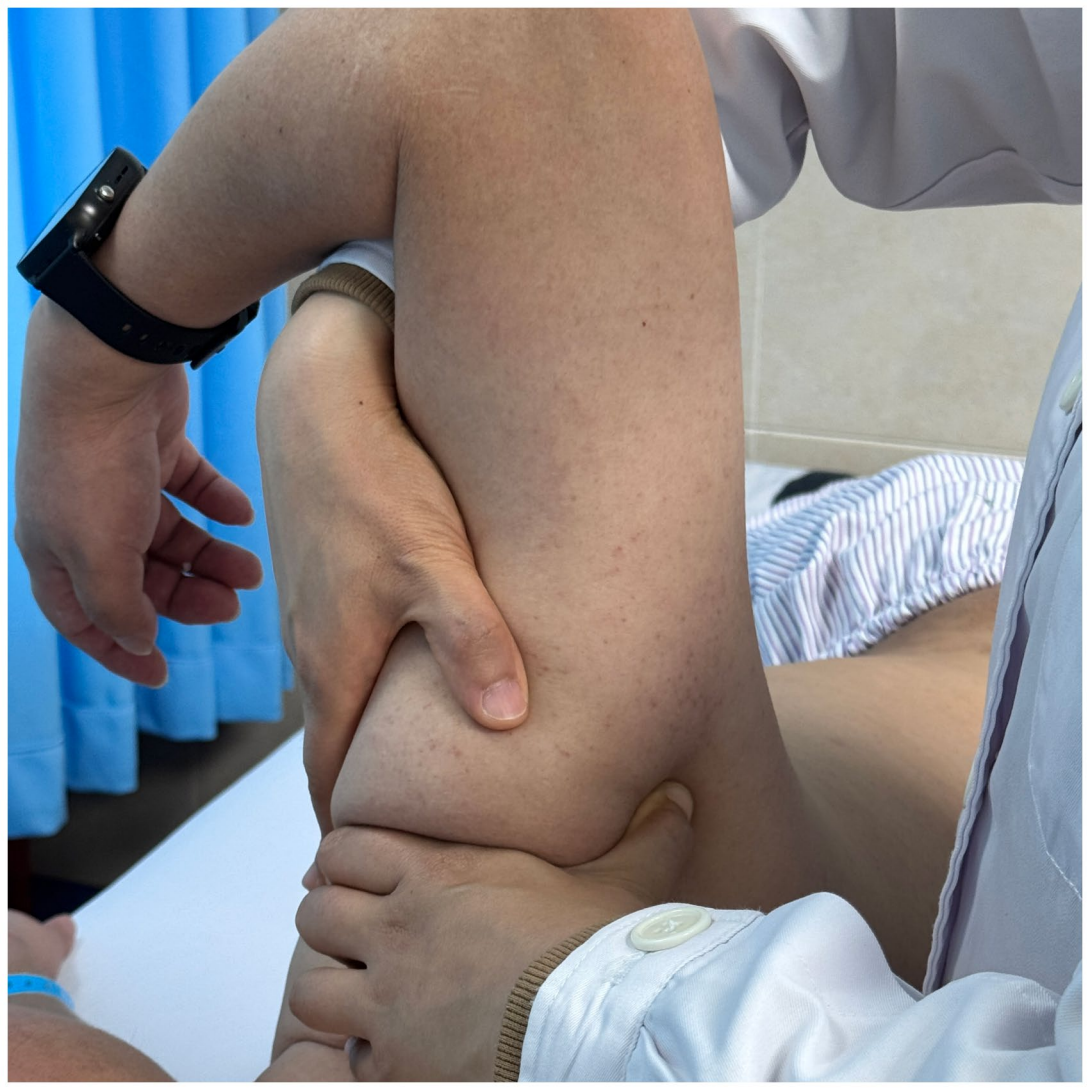
Multidirectional humeral head mobilization therapy.

**Figure 3:**
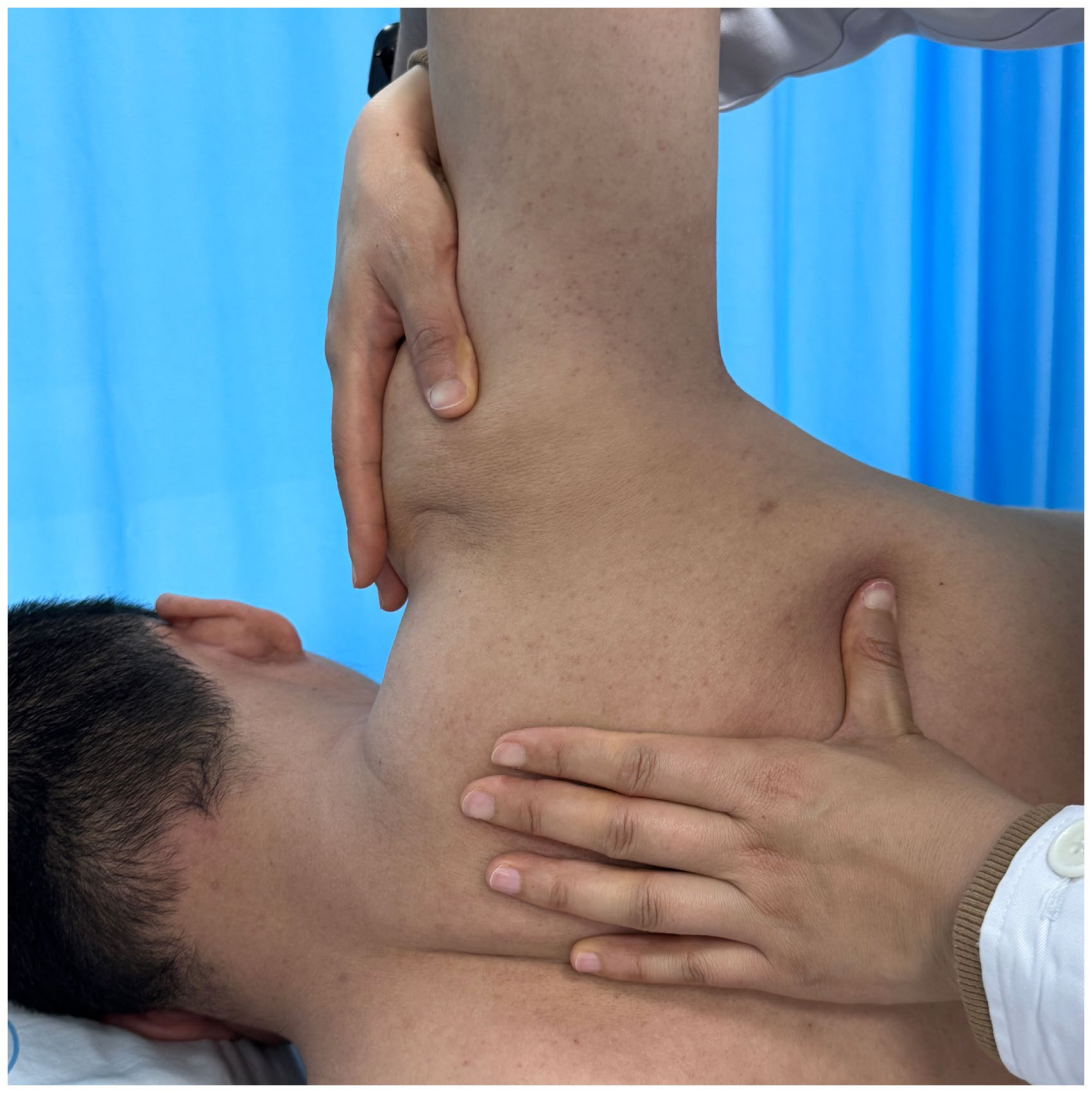
Scapular repositioning.

**Figure 4:**
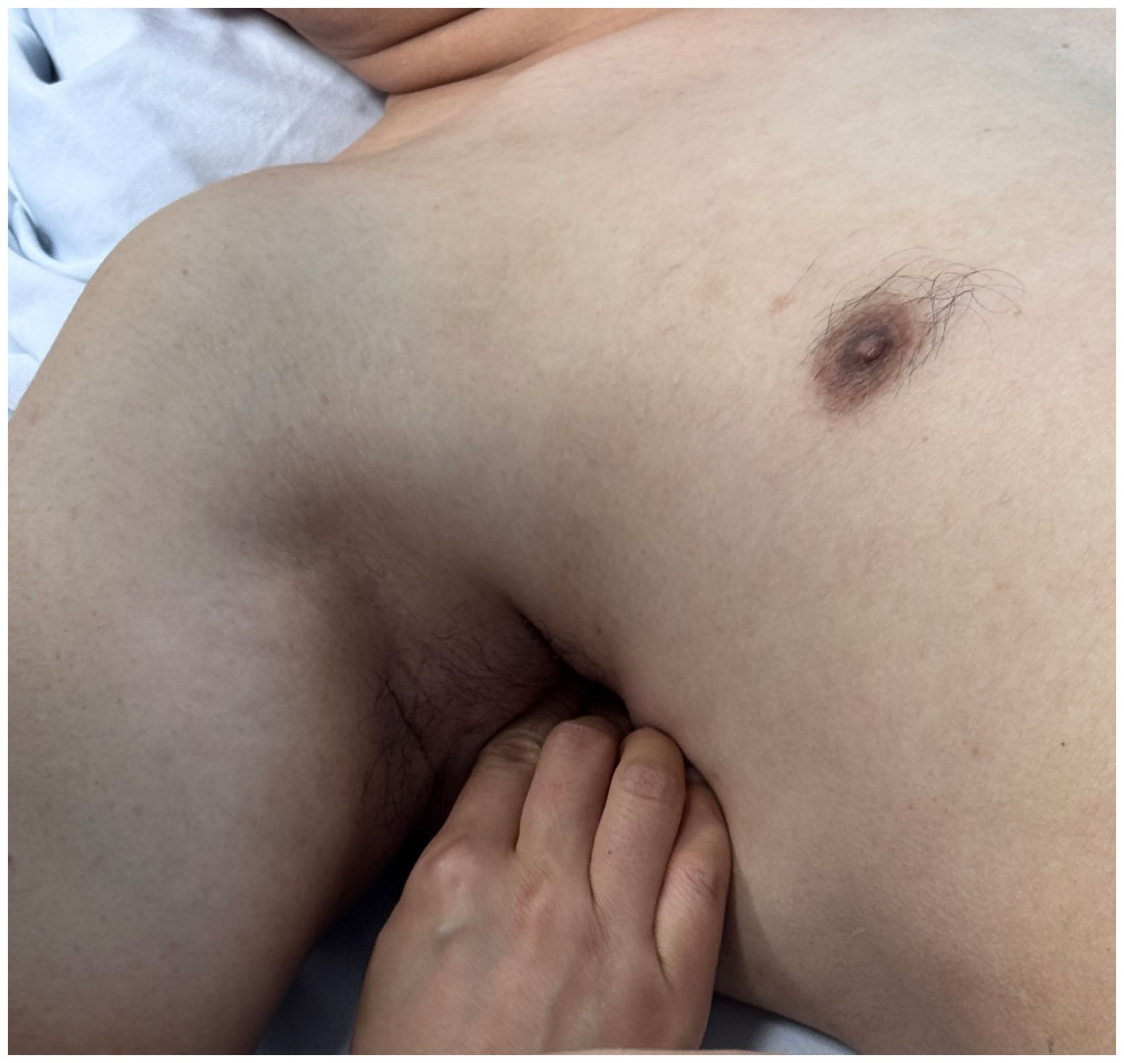
Subscapularis muscle release.

**Figure 5:**
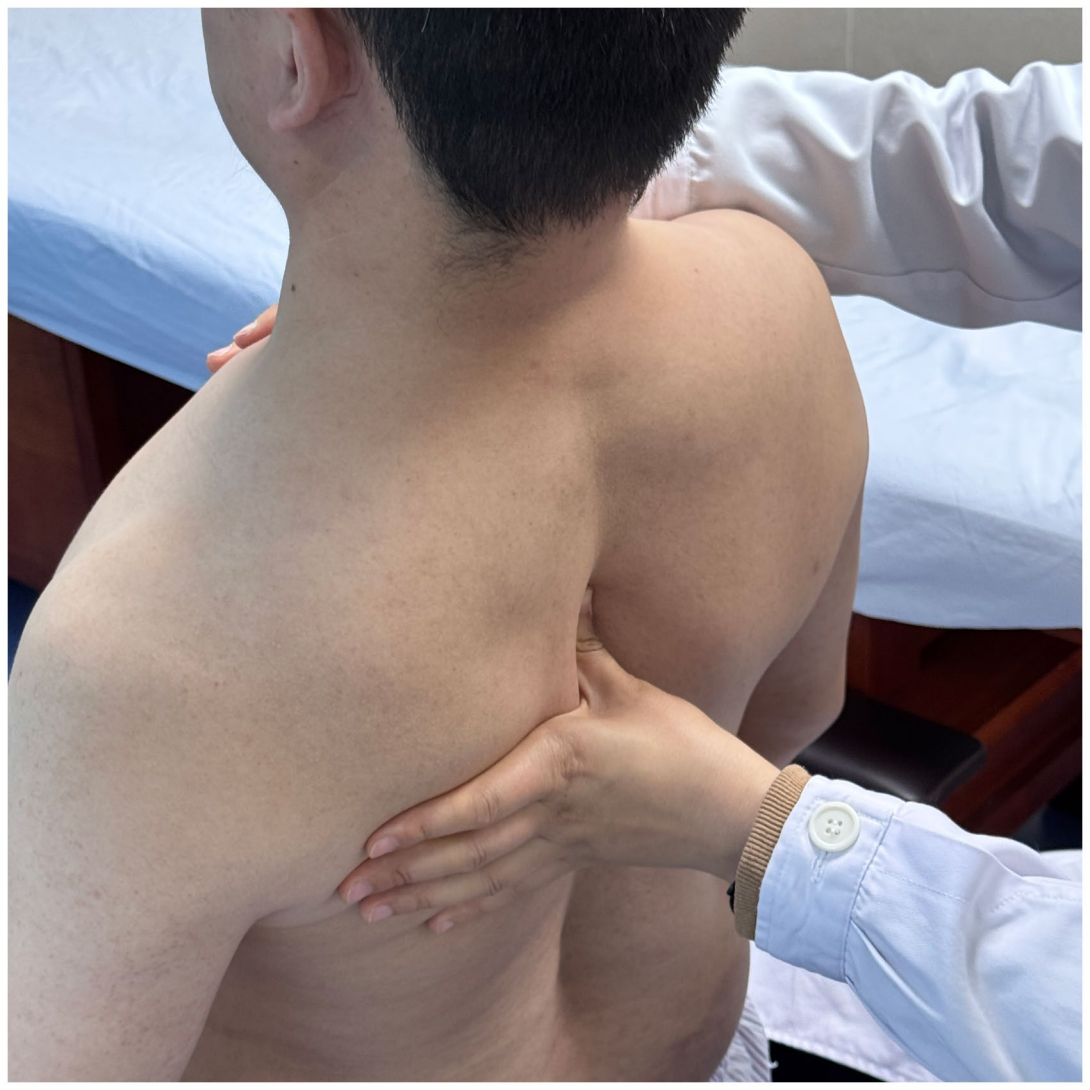
Correction of thoracic kyphosis.

**Figure 6:**
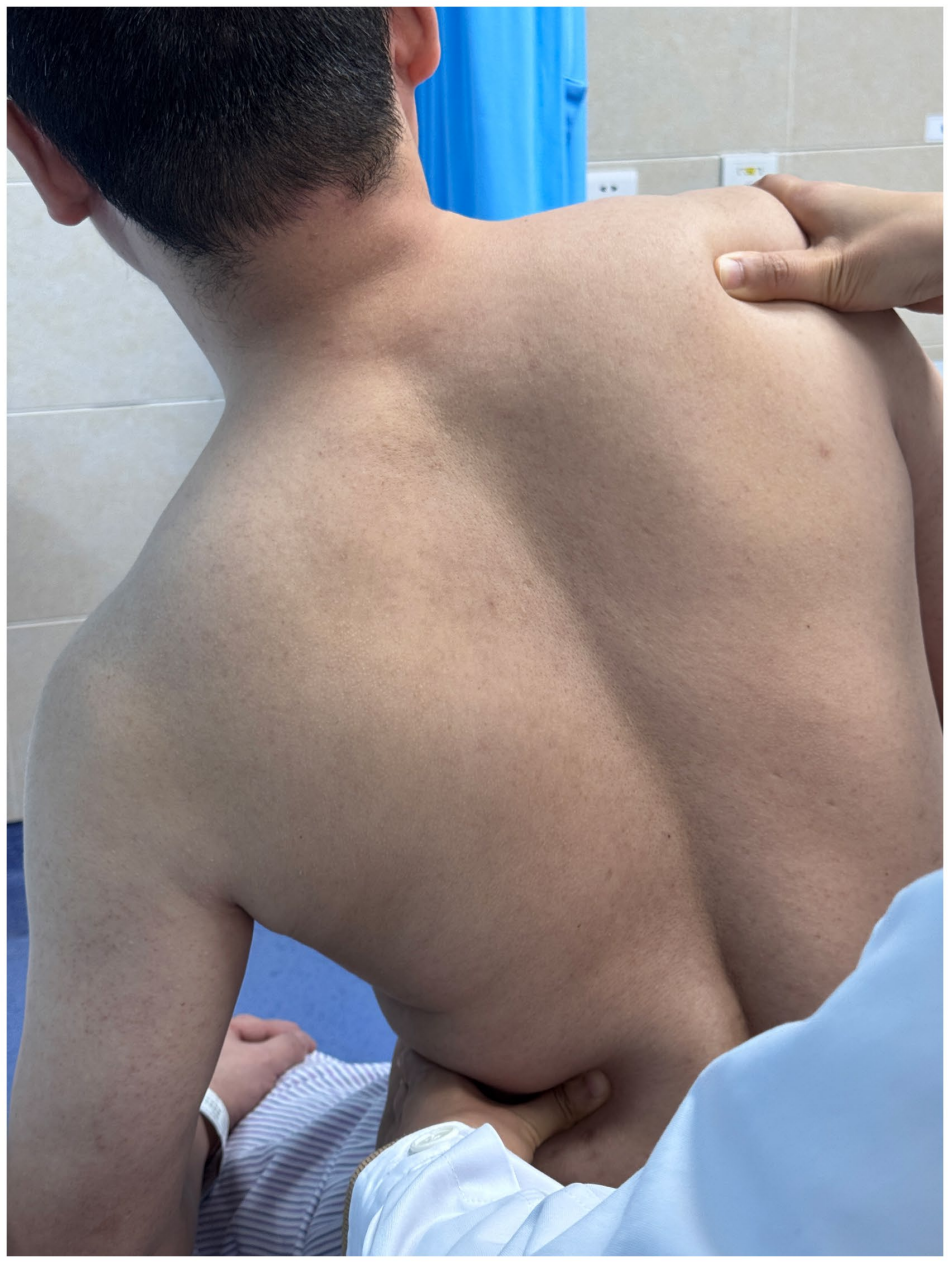
Correction of thoracic kyphosis.

**Figure 7:**
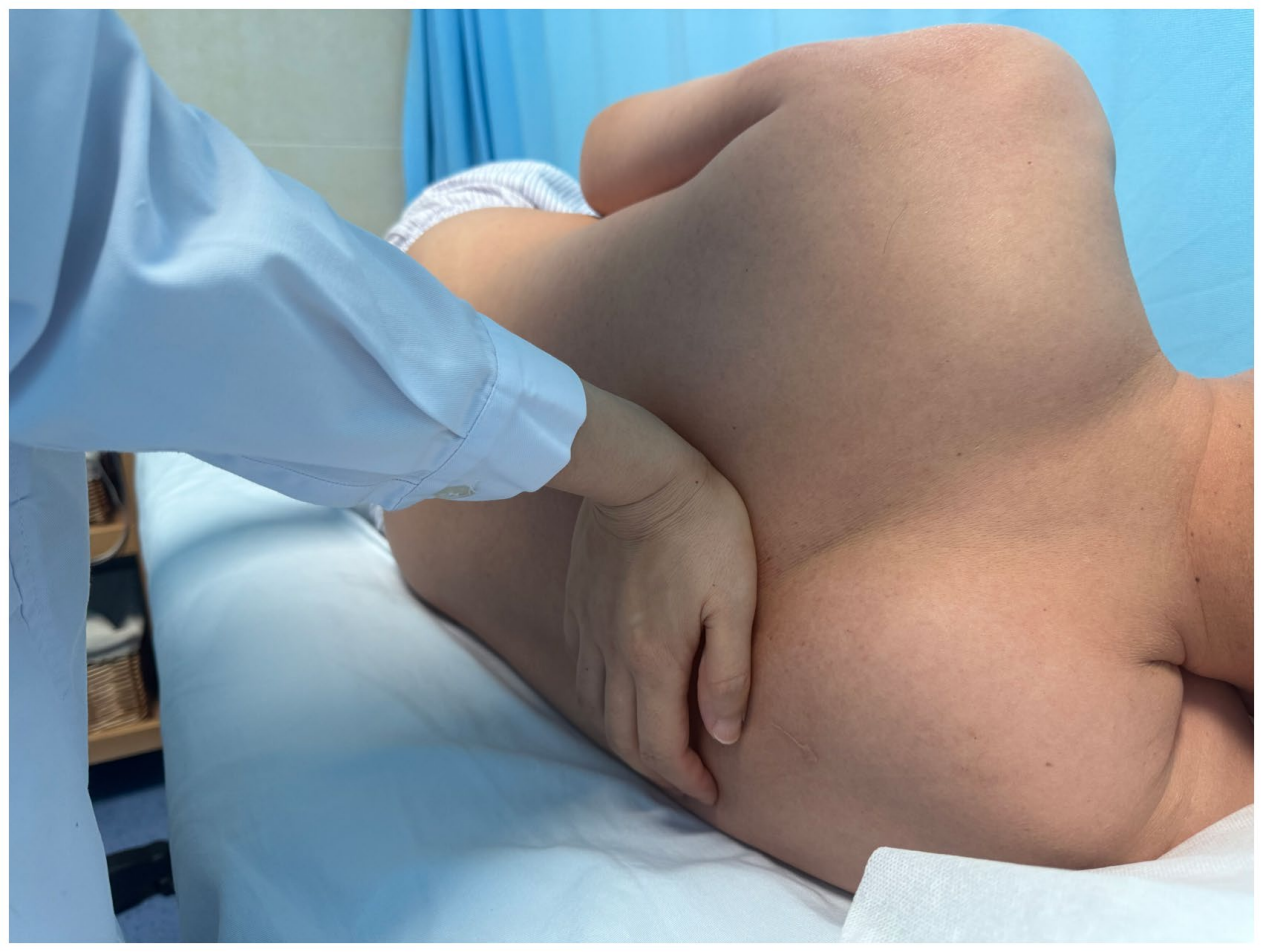
Correction of thoracic transverse process deviation.

Using a patient with right-sided post-stroke shoulder pain as an example, common postural deviations include lumbar spine lateral flexion toward the hemiplegic side, thoracic kyphosis, and scapular depression. The 4R technique is applied to correct these issues. The procedure involves the following steps: (1) With the patient in a seated position, the therapist stabilizes the left lumbar transverse process using one hand, while the other hand wraps around the proximal right upper limb, applying a gentle lateral pull to the left. This is performed 3–5 times. (2) To stretch the right lumbar muscles, including the quadratus lumborum and iliocostalis, the therapist stabilizes the right iliac crest with both hands while the patient actively bends the trunk to the left. The stretch is repeated 3–5 times. (3) The patient then bends the trunk actively to the right end range, holds the position for 8–10 s, and rests before repeating the motion 3–5 times. Once the movement is well controlled, balance disturbance exercises are introduced, such as applying light directional nudges or placing a balance cushion under the pelvis to stimulate proprioceptive response. (4) The therapist mobilizes the T1–T8 spinous processes using posterior-to-anterior techniques, repeated 8–10 times. Additionally, stretches are applied to the platysma, intercostal muscles, and upper rectus abdominis. Each stretch is held for 8–10 s and repeated 3–5 times. (5) Scapular mobilization is performed by stabilizing the axilla with one hand while the other hand mobilizes the inferior medial scapular edge in a medial-to-lateral and upward direction. Simultaneously, the pectoralis minor, subscapularis, and serratus anterior are stretched, each held for 8–10 s and repeated 3–5 times. (6) The humeral head is mobilized in multiple directions, superior, inferior, anterior, posterior, and rotational, within a pain-free range. Each direction is repeated 3–5 times. At the end of each movement, the patient is instructed to hold the final position, with assistance from the therapist if needed. (7) Finally, trigger points around the glenohumeral joint are compressed for 5–10 s, with pressure adjusted based on the patient’s tolerance. Refer to Figures 2–7 for specific procedural illustrations and schematic diagrams.

All treatments are delivered by certified DPS therapists to ensure both safety and accuracy. This structured approach effectively addresses biomechanical abnormalities, relieves post-stroke shoulder pain, and helps restore functional movement patterns.

### Standard Chinese Tuina regimen

SCTT is a widely practiced, non-pharmacological intervention recommended for managing shoulder pain, particularly periarthritis of the shoulder, as outlined in national teaching materials for Traditional Chinese Medicine education during China’s 13th Five-Year Plan (17). SCTT was selected as the control intervention in this study because it represents one of the most commonly applied and guideline-recognized manual therapies in China. In addition, our rehabilitation department is staffed with highly trained therapists who possess extensive clinical experience in tuina, ensuring consistent implementation and high treatment fidelity. These advantages make SCTT a representative and credible comparator for evaluating the clinical effectiveness of the DPS therapy.

In clinical practice, SCTT is typically performed with the patient in a seated position. The therapist begins by identifying painful or dysfunctional regions based on the patient’s reported symptoms and palpation findings. Core techniques such as pressing and kneading are applied to key acupoints, including Jianjing (GB21), Bingfeng (SI12), and Quchi (LI11), for approximately 2–3 min. This is followed by manipulations including kneading, rolling, grasping, and pinching, aimed at releasing muscle tension and stimulating soft tissues in commonly affected areas, particularly the supraspinatus, deltoid, and pectoralis minor muscles. After the initial muscle relaxation phase, treatment proceeds as follows:

1. Muscle Relaxation and Release: The therapist stands on the affected side while the patient remains seated. One hand supports the patient’s upper arm in slight abduction, and the other applies rolling or kneading techniques over the anterior, deltoid, and posterior shoulder areas. Passive movements, abduction, external rotation, and internal rotation, are incorporated to ease spasms and help release adhesions.
2. Spasm Relief and Pain Reduction: Point pressure and plucking techniques are applied to specific acupoints including Jianjing (GB21), Jianyu (LI15), Bingfeng (SI12), Tianzong (SI11), Jianzhen (SI9), Quchi (LI11), Shousanli (LI10), and Hegu (LI4). The therapist aims to produce a sensation of soreness and fullness. Plucking is used on tender or adhesive areas to relieve muscle spasms, reduce pain, and break down adhesions. Each point is stimulated for about 1 min.

The second phase of therapy emphasizes joint mobilization and improving circulation. The therapist begins by stabilizing the shoulder with one hand and using the other to hold the patient’s wrist or elbow, performing circular rotations of the shoulder joint, starting with smaller arcs and gradually increasing the range. Movements include adduction, abduction, extension, and internal rotation. Traction and stretching are applied to help free adhesions and increase joint range of motion. To improve circulation, grasping, rubbing, and pinching techniques are used around the shoulder region. The therapist then gently lifts and stretches the affected arm while applying traction and shaking. Finally, the shoulder, upper arm, and forearm are massaged using rubbing techniques, repeated 3–5 times, to stimulate blood flow and further relax the muscles. All procedures are carried out by licensed and experienced *tuina* therapists to ensure both safety and treatment effectiveness.

### Data analysis

Statistical analyzes will be performed using SPSS version 27.0. All continuous variables will be tested for normality using the Shapiro–Wilk test. Normally distributed data will be expressed as mean ± standard deviation and analyzed using independent sample *t*-tests for between-group comparisons and repeated measures ANOVA for within-group comparisons across time points (baseline, week 2, week 4, and 1-month follow-up). Non-normally distributed data will be reported as medians with interquartile ranges and assessed using the Mann–Whitney U test and Friedman test, respectively. Categorical variables such as gender or stroke type will be analyzed using chi-square tests or Fisher’s exact tests when appropriate. A two-sided *p*-value < 0.05 will be considered statistically significant for all analyzes.

For musculoskeletal ultrasound outcomes, four key indicators will be analyzed: (1) distance from the acromion to the greater tuberosity of the humerus and (2) thickness of the subdeltoid bursa will be treated as continuous variables and analyzed using the same approach as other quantitative measures—independent *t*-tests or Mann–Whitney U tests for between-group comparisons, and repeated measures ANOVA or Friedman tests for within-group changes. (3) Calcifications in the supraspinatus tendon and (4) effusion around the long head of the biceps tendon sheath will be treated as binary variables (presence/absence) and compared between groups using chi-square tests. All ultrasound images will be independently reviewed by two experienced sonographers blinded to group allocation. Inter-rater reliability will be evaluated using Cohen’s kappa for categorical variables and intraclass correlation coefficients (ICC) for continuous variables. Any discrepancies will be resolved by consensus discussion.

## Assessment of outcomes

The primary outcome of this trial is pain intensity measured by the VAS. Secondary outcomes include shoulder range of motion, scapular alignment (vertical distance to T2), musculoskeletal ultrasound findings, and MBI. A detailed summary of these outcome measures and assessment time points is provided in Table 2.

**Table 2:** Overview of outcome measures and assessment time points.

Outcome category	Tool/method	Time points	DPS/SCTT groups
Primary outcome	VAS	Baseline, Week 2, Week 4, Follow-up	√/√
Secondary outcomes	ROM of the shoulder joint	Baseline, Week 2, Week 4	√/√
	Vertical distance (scapula–T2)	Baseline, Week 2, Week 4	√/√
	Musculoskeletal ultrasound	Baseline, Week 2, Week 4	√/√
	MBI	Baseline, Week 2, Week 4	√/√

### Primary outcome

The primary outcome of this study is the change in VAS scores from baseline to 2 weeks, 4 weeks after the intervention, and at the 1-month follow-up. The VAS is a widely used and validated instrument for assessing subjective pain intensity. It consists of a 10 cm (100 mm) continuous line anchored by “no pain” at 0 and “worst imaginable pain” at 10 (18). Patients are instructed to mark the point on the line that best represents their current level of pain. The score is determined by measuring the distance in millimeters from the “no pain” anchor to the patient’s mark. The VAS is known for its sensitivity to changes in pain perception and is commonly used in clinical trials to evaluate the effectiveness of interventions. In this study, it functions as a key indicator of symptom change, providing a measurable outcome for assessing the degree of pain relief in patients with post-stroke shoulder pain (19).

### Secondary outcomes

#### ROM of the shoulder joint

ROM of the shoulder joint reflects its capacity to move in various directions and is an important indicator of functional performance. ROM is typically assessed using a goniometer (3). Normal values include: flexion (lifting the arm forward), 0–180°; extension (moving the arm backward), 0–50°; abduction (lifting the arm to the side), 0–180°; and adduction (bringing the arm toward the trunk), 0–40°. Internal rotation (rotating the arm inward) measures 0–70°, and external rotation (rotating outward) is 0–90°. Horizontal abduction (moving the arm away from the midline in the transverse plane) is 0–45°, and horizontal adduction (bringing it across the body) is 0–135°. These measurements are essential for evaluating joint mobility, identifying functional restrictions, and tracking progress during rehabilitation, particularly in patients with post-stroke shoulder pain.

#### The absolute vertical distance difference between the medial superior angle of the scapula on the affected side and the second thoracic spinous process (T2)

**Figure 8:**
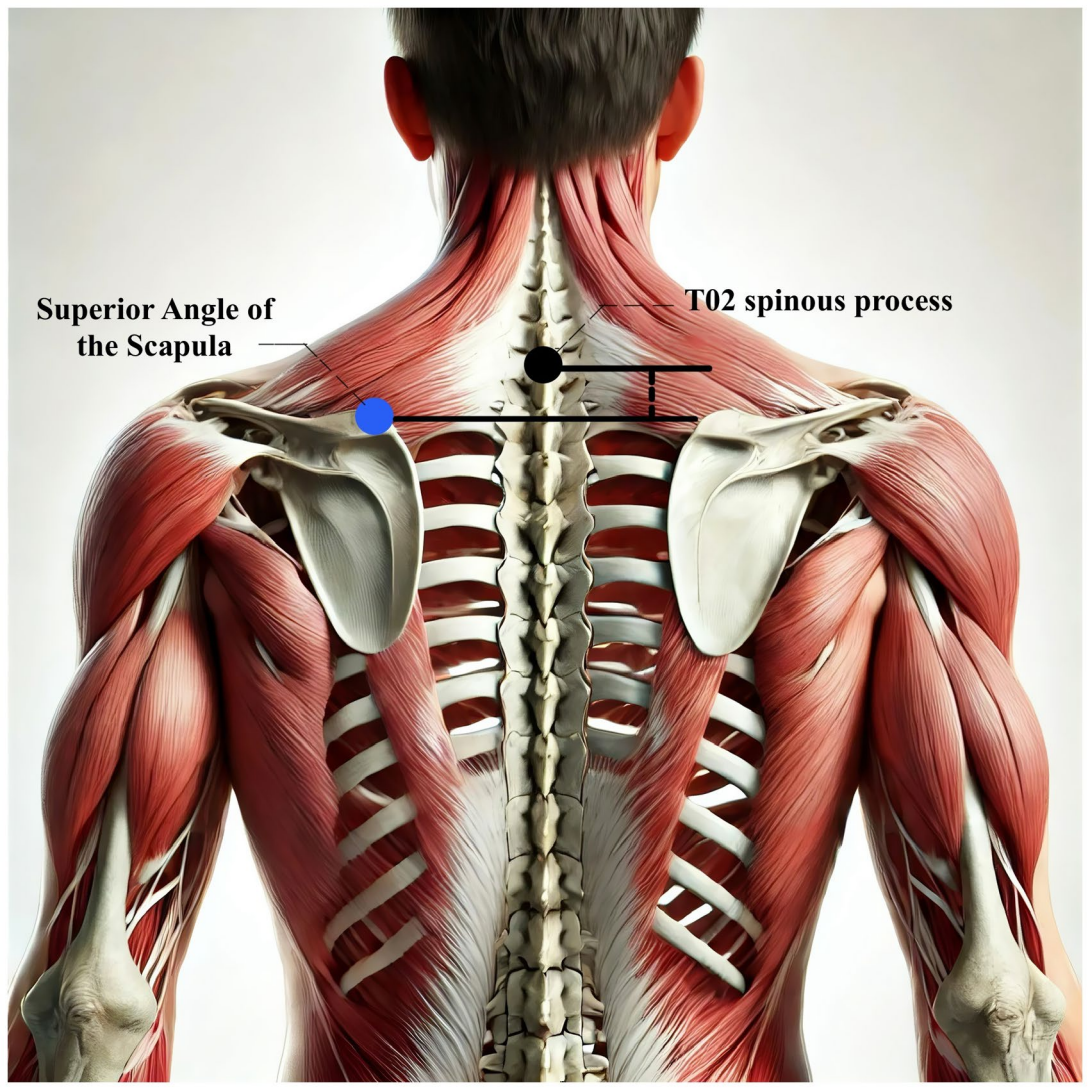
Measurement diagram: medial superior angle of scapula to T2.

This measurement provides an objective assessment of scapular alignment on the affected side. Scapular malalignment is a common feature of PSSP, often resulting from stroke-related muscle imbalance, including weakness or spasticity. Common abnormalities include excessive elevation or downward rotation of the scapula, which can increase mechanical stress on the shoulder joint and contribute to pain and functional deficits. Measuring the vertical distance between the medial superior angle of the scapula and the T2 spinous process allows clinicians to quantify asymmetry and tailor postural correction strategies and therapeutic exercises accordingly (20). A visual reference for the measurement method is provided in Figure 8.

#### Musculoskeletal ultrasound

**Figure 9:**
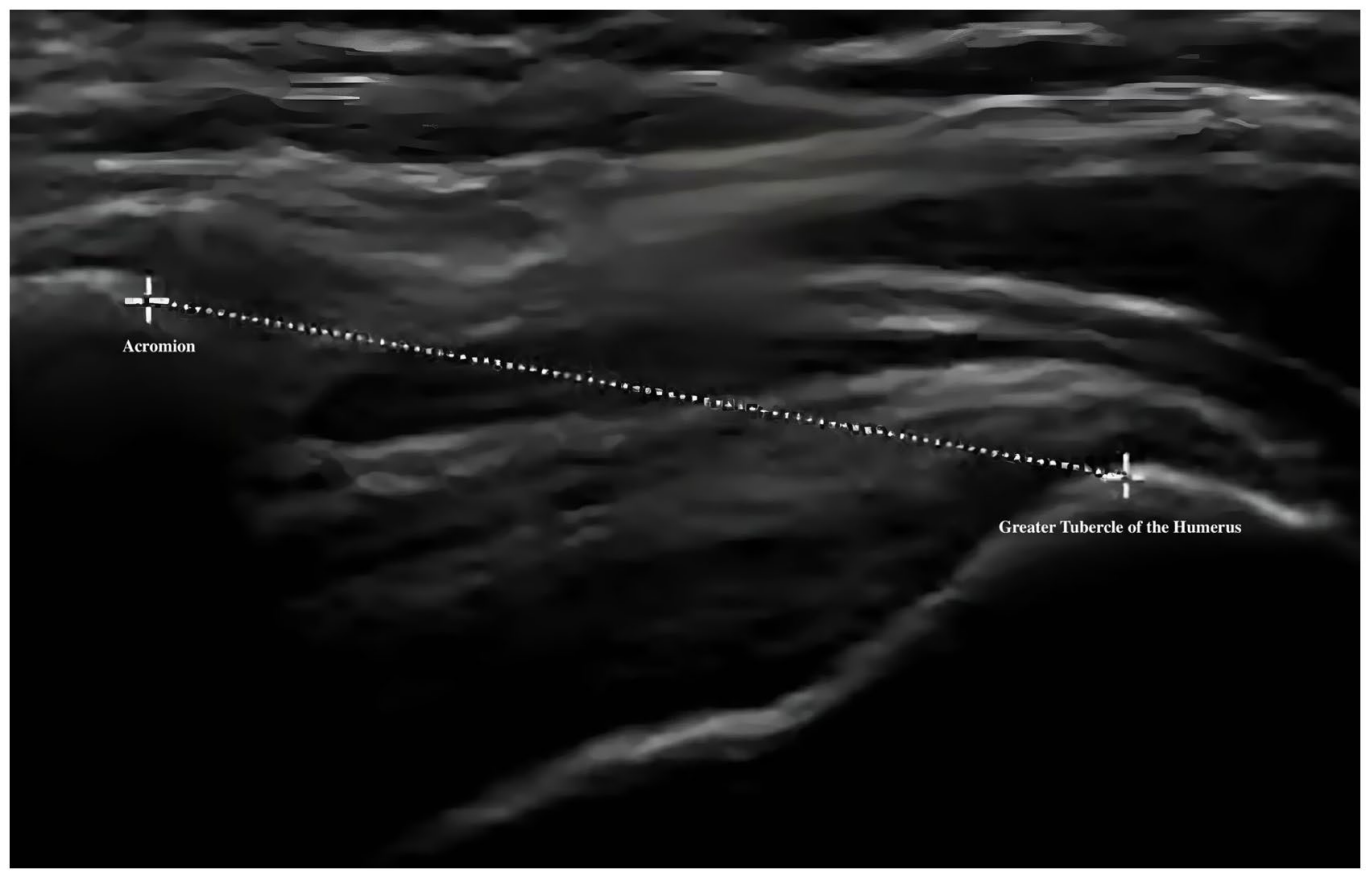
Distance from the acromion to the greater tuberosity of the humerus.

**Figure 10:**
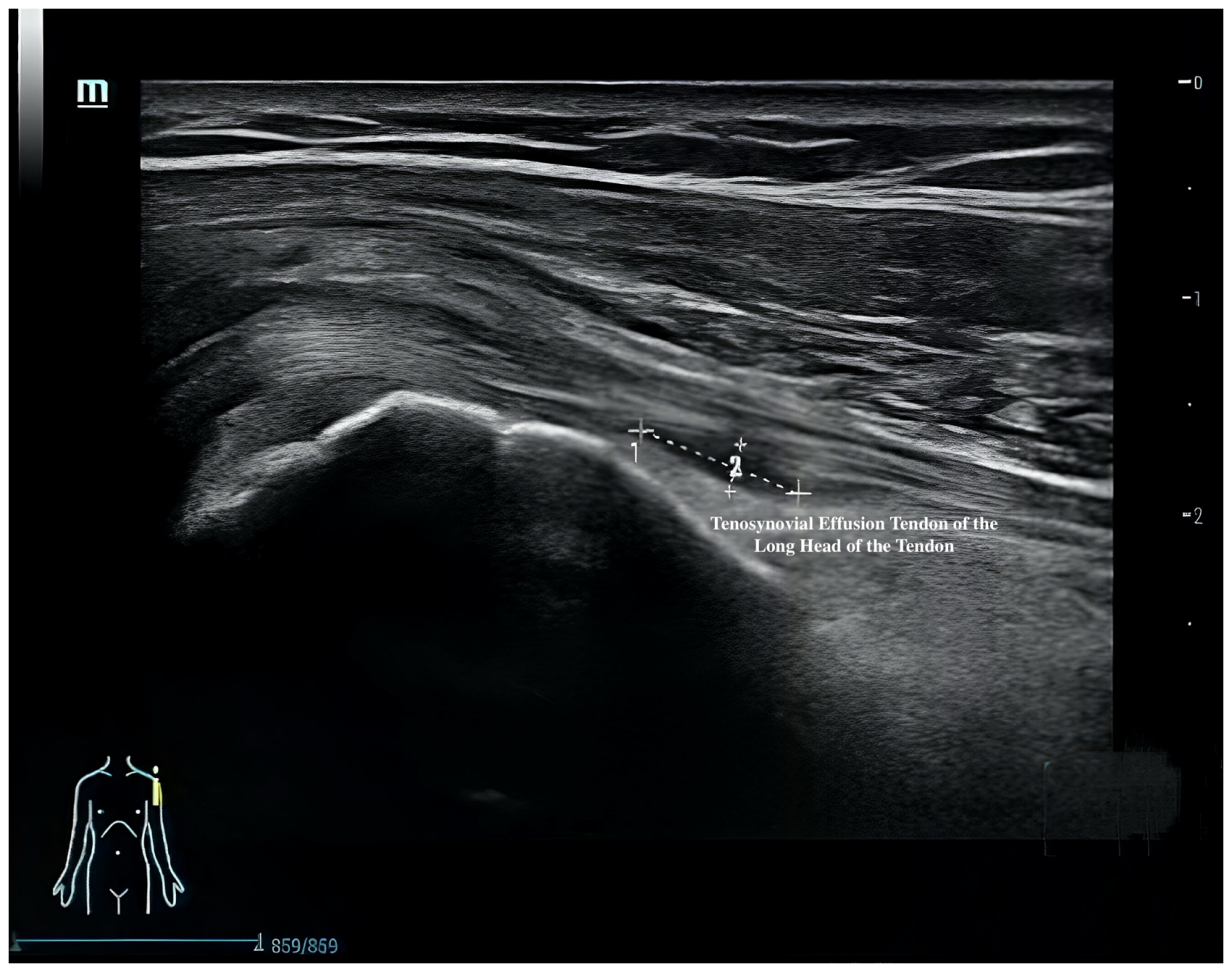
Effusion around the long head of the biceps tendon sheath.

**Figure 11:**
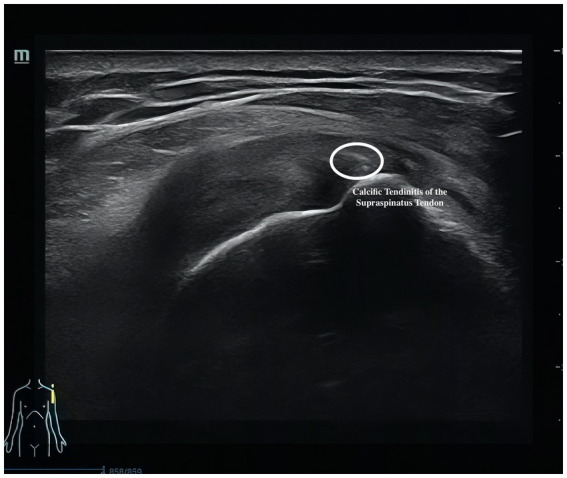
Calcifications in the supraspinatus.

**Figure 12:**
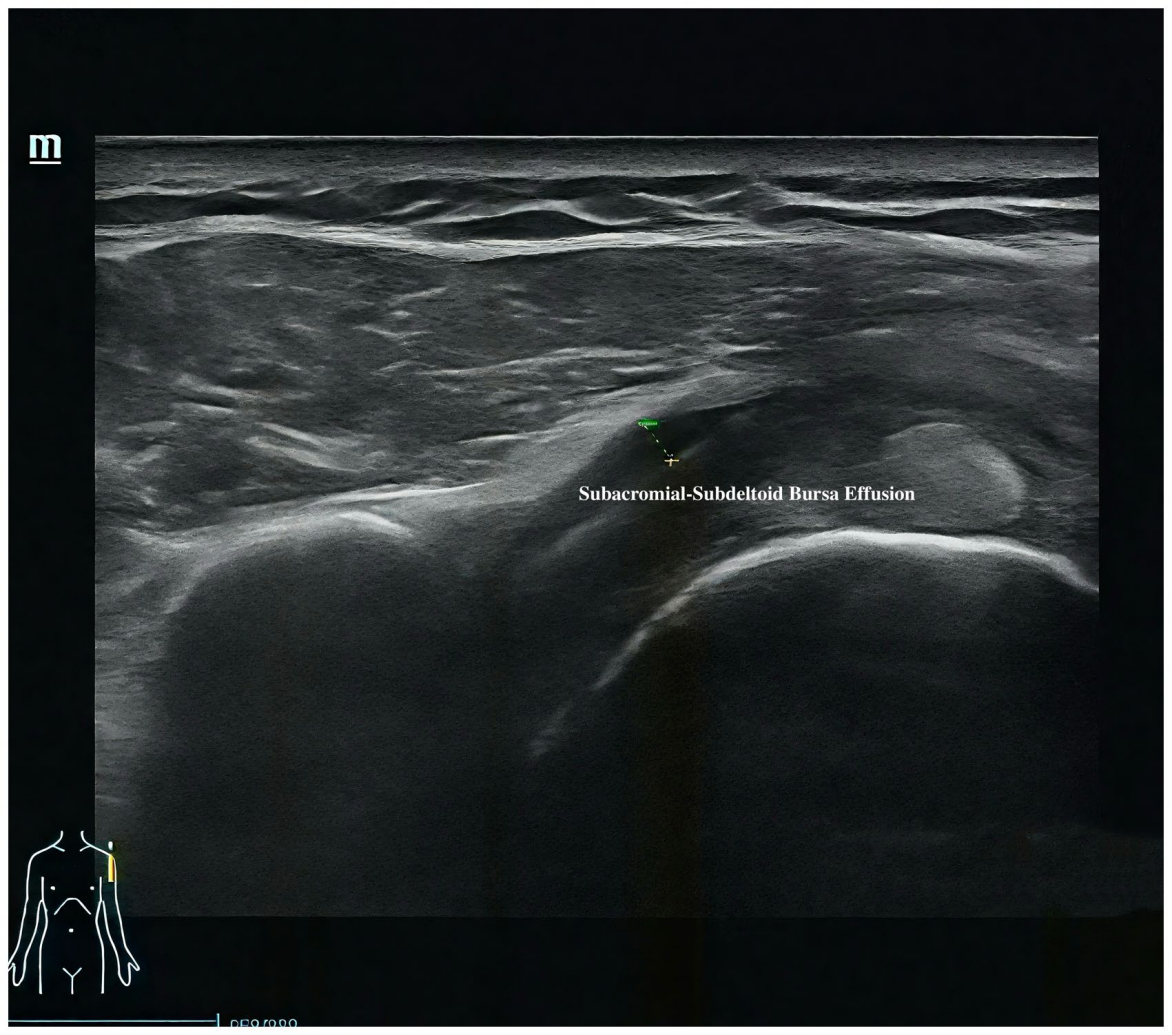
Thickness of the subdeltoid bursa.

Musculoskeletal ultrasound is a practical and noninvasive method for detecting structural abnormalities commonly associated with PSSP. Key indicators include the distance from the acromion to the greater tuberosity of the humerus (Figure 9), effusion around the long head of the biceps tendon sheath (Figure 10), calcification in the supraspinatus (Figure 11) and subscapularis tendons, and increased thickness of the subdeltoid bursa (Figure 12). Effusion around the biceps tendon sheath often reflects inflammation or tendinopathy, which can contribute to pain and limited mobility. Calcifications in the supraspinatus and subscapularis tendons are typically associated with chronic rotator cuff conditions that result in impingement and reduced range of motion. Thickening of the subdeltoid bursa is suggestive of bursitis, a frequent source of discomfort in PSSP patients (21).

Ultrasound examinations will be performed with the patient in either a seated or supine position, depending on the target structure. For the assessment of the long head of the biceps tendon, the patient will be seated facing the examiner with the shoulder in a neutral position and the forearm supinated, resting on the ipsilateral thigh. In the short-axis view, the transducer will be placed transversely over the anterior upper arm and moved proximally to identify the bicipital groove—an osseous depression between the greater and lesser tubercles of the humerus—where the long head of the biceps tendon is located. For the long-axis view, the probe will be rotated 90 degrees after identifying the tendon in the short axis. Scanning will continue distally to the musculotendinous junction and proximally into the glenohumeral joint space.

For the subscapularis tendon, the patient will externally rotate the arm to expose the tendon. From the short-axis view of the biceps tendon, the probe is moved medially into the lesser tuberosity region to obtain the long-axis view of the subscapularis tendon, which appears beak-shaped at its humeral insertion. Passive internal and external rotation of the shoulder may be used to dynamically assess the integrity of the tendon. For the short-axis view of the subscapularis, the probe is rotated 90 degrees from the long axis. The tendon may appear as multiple fascicles interspersed with hypoechoic muscle tissue; a comprehensive medial-to-lateral sweep will be performed to evaluate the entire tendon.

For supraspinatus tendon evaluation, a modified Crass position will be used. The patient will extend the shoulder posteriorly, flex the elbow, and place the dorsal hand over the posterior iliac wing on the ipsilateral side—roughly over the back pocket area. This positioning rotates the supraspinatus tendon anteriorly, allowing sagittal imaging over the greater tuberosity. The tendon will first be examined in the long-axis view, followed by a 90-degree probe rotation to visualize the short-axis view. The presence of calcific deposits will be recorded. The thickness of the subacromial-subdeltoid (SASD) bursa will be measured at its thickest point, and care will be taken to avoid excessive transducer pressure. In the neutral seated position with the arm relaxed at the side, the vertical distance between the acromion and the greater tuberosity of the humerus will also be measured.

For patients with limited mobility or severe pain, the standard Crass position may be modified to ensure patient comfort. In the traditional Crass position, the patient places the dorsum of the hand behind the back to touch the contralateral scapula, which helps tension the supraspinatus tendon and may reveal subtle tears. However, this posture may exacerbate discomfort in symptomatic patients and will be used cautiously.

Each ultrasound scan is conducted by two musculoskeletal ultrasound specialists who independently evaluate the images using a blinded assessment protocol. If both assessments align, the result is confirmed. In case of disagreement, the specialists discuss the findings to reach a consensus. If they agree following the discussion, the shared conclusion is accepted. If consensus cannot be reached, the sample is excluded and not used in the final analysis.

#### MBI

The MBI is a widely used scale for evaluating functional independence in activities of daily living (ADLs) in post-stroke populations. In patients with PSSP, MBI is especially relevant, as shoulder pain can significantly interfere with daily tasks such as grooming, feeding, and dressing. Lower MBI scores have been linked to higher levels of dependence and poorer functional outcomes in individuals experiencing post-stroke shoulder pain (22, 23). Research shows that PSSP often limits mobility and hampers basic self-care, with particular effects on dressing and walking. Monitoring MBI scores in these patients allows clinicians to assess the impact of pain on daily function and adjust rehabilitation plans accordingly to improve outcomes.

### Adherence

To support adherence and improve rehabilitation outcomes in patients with post-stroke shoulder pain, several structured measures are implemented during hospitalization: (1) A strict ward management system is maintained. All patients are required to stay within the rehabilitation ward to prevent interruptions in therapy, ensure continuity of care, and allow immediate medical response in case of complications or emergencies. (2) A daily attendance system is in place. Patients use a rehabilitation treatment card to check in for each session, which therapists sign after completion to verify participation. (3) Nurses play a key role in reinforcing adherence. They provide bedside education, distribute printed materials, and share instructional videos to help patients understand the significance of rehabilitation. Daily adherence checks are also conducted, during which nurses speak with patients to identify obstacles and suggest practical solutions. (4) The rehabilitation team delivers individualized multidisciplinary support. Physicians create tailored rehabilitation plans; therapists adjust treatment intensity based on patient tolerance; and nurses monitor progress closely. A weekly multidisciplinary meeting is held to evaluate each patient’s participation and make any necessary adjustments to their care plan. (5) Psychological support is integrated to maintain motivation and reduce resistance to therapy. Medical staff offer encouragement, share successful case examples, and involve family members for emotional reinforcement. For patients with low engagement, motivational interviewing techniques are applied to explore concerns, stress the importance of rehabilitation, and build confidence in the recovery process. Through this coordinated strategy, including ward restrictions, attendance tracking, regular monitoring, individualized care, and psychological support, patient adherence is actively reinforced, which contributes to better rehabilitation outcomes and higher standards of care.

### Safety

Throughout the intervention period, adverse events will be monitored weekly. An adverse event is defined as any unwanted experience occurring during the study. All such events will be recorded in the Adverse Event Case Report Form (CRF) to ensure consistent documentation and analysis.

## Discussion

This study presents a rigorously designed randomized controlled trial protocol comparing the efficacy of Discovery of Posture Secret manual therapy with Standardized Chinese Tuina Therapy in patients with PSSP. As a common and functionally disabling complication in stroke survivors, PSSP remains a challenge to effective rehabilitation. The trial’s design integrates both subjective and objective outcome assessments, with a particular emphasis on incorporating musculoskeletal ultrasound to enhance the objectivity and clinical relevance of the evaluation process. The combination of a novel intervention approach and a multimodal outcome framework represents a comprehensive strategy to address this complex condition.

While SCTT remains a widely practiced and guideline-supported therapy for shoulder pain in China, it is primarily focused on local muscle relaxation and relies heavily on therapist experience. In contrast, DPS introduces a structured, biomechanically informed manual therapy model aimed at restoring joint alignment, reactivating neuromuscular control, and enhancing postural stability. By systematically addressing joint malalignment, abnormal muscle recruitment, and proprioceptive deficits, DPS is expected to offer broader and more sustained functional benefits compared to conventional tuina. The structured nature of DPS may also contribute to improved reproducibility and standardization in clinical practice, a longstanding challenge in traditional manual therapy.

The outcome framework of this trial includes one primary and several secondary measures, designed to reflect both patient-reported symptoms and functional recovery. Pain intensity, measured by the VAS, serves as the primary outcome due to its direct relevance to patient experience and its sensitivity to change. Secondary outcomes such as shoulder joint ROM, scapular alignment (quantified by the vertical distance to the T2 spinous process), musculoskeletal ultrasound, and the MBI are essential for capturing improvements in joint function and daily living capacity. Together, these indicators provide a multifaceted understanding of how different therapeutic strategies impact recovery.

A notable innovation in this study is the use of musculoskeletal ultrasound as an objective and structural assessment tool. Traditional evaluations of PSSP have largely relied on subjective scales or clinical observation, often lacking precise anatomical insight. In contrast, musculoskeletal ultrasound enables visualization and quantification of structural abnormalities such as subdeltoid bursa thickening, calcifications in the rotator cuff tendons, effusion around the long head of the biceps tendon, and reduced acromion-to-greater tuberosity distance. These structural changes are closely linked to pain and dysfunction in PSSP, and their inclusion in the outcome set allows for a more granular evaluation of treatment effects. Moreover, blinded dual-sonographer image assessment and the use of inter-rater reliability analysis contribute to the scientific rigor and reliability of these imaging outcomes.

Beyond its role as a diagnostic and monitoring tool, musculoskeletal ultrasound may also provide insight into the underlying mechanisms through which manual therapies exert their effects. For instance, a reduction in bursal thickness or tendon effusion following DPS intervention may suggest not only symptom relief but also a genuine reversal of soft tissue inflammation or mechanical impingement (24). In this way, ultrasound imaging serves not only as a clinical endpoint but also as a potential biomarker of therapeutic response, paving the way for mechanism-based rehabilitation research.

From a biomechanical perspective, DPS offers a compelling theoretical framework for addressing the pathophysiology of PSSP. Abnormal postural patterns and joint malalignment—such as thoracic rotation, scapular depression, or glenohumeral instability—are common in stroke survivors due to neuromuscular imbalance and compensatory movement (19). The four core components of DPS—Resetting Joint Malalignment, Resetting Abnormal Muscle Function, Resetting Joint Stabilization, and Resetting Sensory-Motor Control—are designed to systematically correct these dysfunctions. By restoring normal scapulothoracic and glenohumeral relationships, DPS may reduce secondary impingement, redistribute mechanical loads, and re-establish efficient motor patterns. The integration of biomechanical correction with sensorimotor retraining is particularly relevant in stroke rehabilitation, where central and peripheral impairments often coexist.

Despite the methodological strengths of this study, several limitations must be acknowledged. As a single-center trial with a modest sample size and relatively short follow-up period, the generalizability of the findings may be limited. Although ultrasound provides objective structural information, its interpretation remains somewhat operator-dependent despite efforts to standardize procedures. Future studies should explore the use of dynamic ultrasound to further elucidate the biomechanical mechanisms underlying therapeutic response. Additionally, long-term multicenter trials are needed to evaluate the durability of DPS outcomes and to validate its application across different PSSP subtypes and patient populations.

In summary, this study protocol combines a novel biomechanical manual therapy with objective imaging-based outcome measures to explore a comprehensive treatment strategy for post-stroke shoulder pain. The integration of musculoskeletal ultrasound enhances methodological rigor and provides a structural window into treatment response, while the biomechanical foundation of DPS contributes to a deeper understanding of rehabilitation mechanisms. This trial is expected to inform both clinical practice and future research in post-stroke rehabilitation.
